# Polyamines: Small Amines with Large Effects on Plant Abiotic Stress Tolerance

**DOI:** 10.3390/cells9112373

**Published:** 2020-10-29

**Authors:** Rubén Alcázar, Milagros Bueno, Antonio F. Tiburcio

**Affiliations:** 1 Polyamine’s Laboratory, Department of Biology, Healthcare and Environment, Faculty of Pharmacy and Food Sciences, University of Barcelona, 08028 Barcelona, Spain; ralcazar@ub.edu; 2 Laboratory of Plant Physiology, Department of Animal Biology, Plant Biology and Ecology, Faculty of Experimental Science, University of Jaén, 23071 Jaén, Spain; mbueno@ujaen.es

**Keywords:** drought, salinity, heat, cold, putrescine, spermidine, spermine, thermospermine, climate change, plant stress

## Abstract

In recent years, climate change has altered many ecosystems due to a combination of frequent droughts, irregular precipitation, increasingly salinized areas and high temperatures. These environmental changes have also caused a decline in crop yield worldwide. Therefore, there is an urgent need to fully understand the plant responses to abiotic stress and to apply the acquired knowledge to improve stress tolerance in crop plants. The accumulation of polyamines (PAs) in response to many abiotic stresses is one of the most remarkable plant metabolic responses. In this review, we provide an update about the most significant achievements improving plant tolerance to drought, salinity, low and high temperature stresses by exogenous application of PAs or genetic manipulation of endogenous PA levels. We also provide some clues about possible mechanisms underlying PA functions, as well as known cross-talks with other stress signaling pathways. Finally, we discuss about the possible use of PAs for seed priming to induce abiotic stress tolerance in agricultural valuable crop plants.

## 1. Introduction

The ever-increasing human population, together with the loss of agricultural land (due to urbanization, industrialization, desertification and climatic changes) as well as the diminishing availability of resources, poses serious challenges to the world agriculture. There is substantial evidence that climate change is leading to an increase in the frequency and intensity of extreme climate events such as heat waves, severe drought periods, as well as extreme precipitation and storms [1,2,3]. Thus, drought, salinity and high and low temperatures extremes are among the major stresses that adversely affect plant growth and productivity worldwide. In particular, these events are important for Europe, since four out of the five worldwide extreme heat wave events that have occurred within the last 15 years were observed in European regions [4]. Water deficiency negatively impacts plant growth and productivity, and research efforts are focused on the development of strategies to mitigate its effects on crop yield by selection of drought-adapted varieties and/or by improving soil management and irrigation techniques [5]. Land degradation due to soil salinization is also a serious problem, the impact of which increases steadily in many parts of the world, especially in arid and semiarid regions. In fact, most crops species used in agriculture are glycophytes (salt sensitives) and, therefore, their productivity is being severely affected in many regions of the world [6]. Temperature is one of the most important environmental factors limiting the geographical distribution of plants and accounts for significant reductions in the yield of agriculturally important crops. Temperature above or below a certain range has a negative impact on plant performance, which leads to a loss in economic yield. Chilling and freezing stresses are collectively known as cold or low-temperature stress [7]. Temperatures above the optimum range for plant growth and development, which are defined as heat stress, can damage both vegetative and reproductive organs of plants [8].

Under water deficit and/or salinity conditions, as well as under cold and heat, plants initiate a number of physiological and metabolic responses, which are orchestrated by post-translational modifications and changes in gene expression [9]. Many plant genes involved in abiotic stress responses have been identified. Among them are genes controlling the synthesis of protective metabolites like osmolytes and PAs [10]. Indeed, elevated PA levels are one of the most remarkable metabolic hallmarks in plants exposed to abiotic stress conditions such as drought, salinity, chilling, heat, hypoxia, ozone, UV, heavy metals and herbicides. These changes are mainly produced by alterations in PA metabolism and/or interactions with other metabolic and/or signaling pathways in response to stress [10,11,12]. Polyamines are beneficial for protein homeostasis, detoxification of reactive oxygen species (ROS), activation of the antioxidative machinery, and molecular chaperone activity under stress conditions, thereby providing broad-spectrum tolerance against a variety of stresses [13].

**Figure 1:**
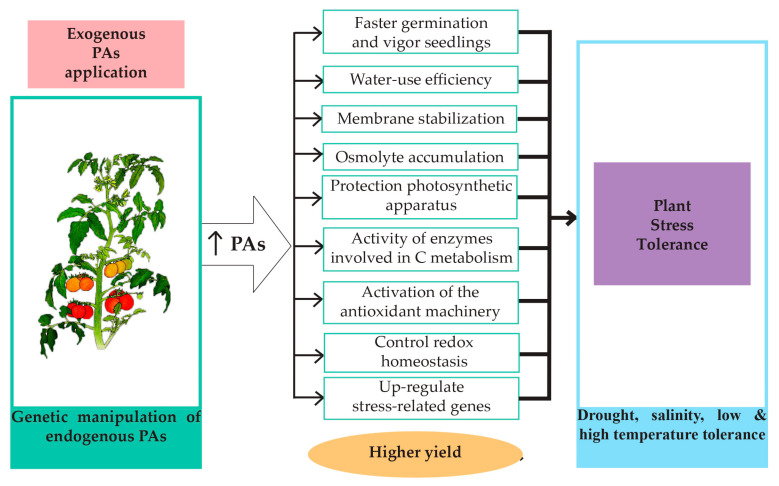
Polyamine accumulation triggers several molecular, biochemical and physiological responses that promote stress tolerance, thus increasing crop yield.

Evidence indicates that exogenous applications of PAs, mainly putrescine (Put), spermidine (Spd) and spermine (Spm), protects against the damage induced by different types of abiotic stresses [14,15]. Chen et al. [15] reviewed the relationship between PAs and flowering time, embryo development, senescence and response to several abiotic stresses. The review highlighted the protective mechanism of the exogenous PA application in a great variety of agricultural crops against water, saline and temperature stress. In this review, we deepen the knowledge about the involvement of PAs in abiotic stress in additional crops, and discuss seed priming as an efficient and straightforward mechanism to induce crop tolerance using exogenous PAs. On the other hand, recent transcriptomic studies performed in plants (i.e., *Arabidopsis*) have revealed the differential regulation of PA metabolism genes during abiotic stress. The characterization of PA loss-of-function mutants in this species has provided evidence for the involvement of these compounds in stress-resistance traits. Overexpression of several PA biosynthetic genes from different organisms in many plant species has been shown to induce stress tolerance in correlation with the degree of PA accumulation [10,12,16,17,18]. The use of PA engineering for crop protection has also been discussed. Compelling evidence thus indicates that PAs have a protective role against abiotic stress in different plant species (Figure 1).

## 2. Polyamines and Drought Stress

Drought can be defined as a period of abnormally dry weather, long enough to cause serious effects on water balances. Drought is one of the most adverse factors for plant growth and productivity, and it is considered a severe threat for sustainable crop production in the context of climate change [19,20]. Plants’ responses to drought include morphological, physiological, molecular, hormonal and transcriptional changes, which are intricately coordinated and depend on the duration and severity of the water limitation. Following drought, stomata close progressively with a parallel decline in net photosynthesis and water-use efficiency. In addition to other factors, changes in photosynthetic pigments are required for drought tolerance. Protective responses in leaves must also be executed to prevent damage of the photosynthetic machinery. ROS scavenging by enzymatic and non-enzymatic antioxidant systems, cell membrane stability, expression of aquaporines and stress proteins, ion balance, and accumulation of osmolytes and osmoprotectants represent essential mechanisms contributing to drought tolerance. Furthermore, genetic variation and developmental stage (being germination and flowering, the most sensitive stages to stress) also influence the final outcome of drought stress [20,21].

### 2.1. Protective Effects by Exogenous Application of PAs during Drought 

The effects on plant tolerance induced by exogenous PA applications are summarized in Table 1, Table 2 and Table 3, in which techniques for PA application and stress treatments are detailed. Under drought conditions, exogenous PA treatments lead to improved stress tolerance in the various plants species studied [14]. For example, exogenous application of Put by foliar sprayings enhanced water status, chlorophyll, proline (Pro), amino acids and soluble sugars contents in wheat plants subjected to water stress, which resulted in enhanced plant height, leaf area and grain yield [22] (Table 1). Tobacco leaf discs pretreated with Put one hour before polyethylene-glycol addition significantly prevented water loss and maintained the maximum photochemical efficiency of photosystem II, suggesting an important role for Put in the modulation of plant tolerance against osmotic stress [23] (Table 1). One of the best plant responses against drought is to keep water content in tissues. Recently, Zhu et al. [24] observed that exogenous Put application (foliar sprayings) reduced stomatal density, maintained the chloroplast structure, and prevented cell plasmolysis, which contributed to an increase in water-use efficiency and drought tolerance in lettuce.

In relation to higher molecular weight PAs, pretreatment with Spd in bermudagrass plants resulted in greener leaf tissue and increased survival rate after drought or salinity stresses in comparison with untreated plants [25] (Table 1). Physiological and proteomic analyses suggested that PAs could activate multiple pathways (including proteins involved in electron transport and energy, and antioxidant enzyme systems), as well as osmolyte accumulation that enhances bermudagrass adaptation to drought and salt stresses. In agreement with this, spraying valerian plant with Spd or Spm also increased the activities of antioxidant enzyme-systems, Pro content, and photosynthetic pigments in response to drought stress [26]. Pretreatment with Spm conferred dehydration tolerance of citrus plants cultured in vitro via modulation of antioxidative capacity and stomatal closure [27] (Table 1). Water-stressed finger millet (*Eleusine coracana* L. Gaertn.) plants sprayed with 0.2 mM Spd at early flowering stage, showed protection against chlorophyll degradation, and produced less electrolyte leakage, lower levels of hydrogen peroxide (H_2_O_2_), and caspase-like activity than unstressed plants, as well as accumulation of Pro alleviating the water deficit [28]. Another example was noted in Damask rose in which foliar applications of Spm or Spd (0.5 mM) improved relative water and chlorophyll contents, as well as stomatal conductance in plants subjected to water stress. The levels of Pro and antioxidant enzyme activities were also increased in rose plants. The exogenous application of Spm or Spd produced the activation of the antioxidant machinery and changes in PA metabolism, resulting in increased tolerance to water stress [29]. 

Seed germination and early seedling growth are the most sensitive stages to water deficit. Drought induces a reduction in germination rate and a delay in the initiation of germination and seedling establishment [30]. Therefore, it is important to establish suitable approaches that might alleviate the negative effects on seed germination caused by water deficit. Seed priming is a pre-sowing treatment that exposes seeds to a certain solution that allows partial hydration, but radicle emergence does not occur [31]. Priming allows the initiation of many physiological processes associated with the early phase of germination but prevents transition to full germination [31]. There are several priming techniques, such as hydropriming, osmopriming, halopriming, or chemical priming, which are the most commonly used [32]. After removal of seeds from the priming solution, seeds are re-dried to the initial moisture content, thus maintaining the beneficial effects of the priming treatment without loss of quality caused by rapid seed deterioration [31]. When the primed seeds are sown, the swelling of the embryos, inside the seeds, speeds up germination by facilitating water absorption. It stimulates pre-germination metabolic processes, so that the seedlings emerge faster, grow more vigorously, and perform better under adverse conditions, thus protecting seeds from abiotic and biotic stresses during the critical phase of seedling establishment [31]. Chemical priming is a promising field in plant stress physiology and crop stress management [32]. Polyamines are among the group of chemicals acting as priming agents that can potentially confer enhanced tolerance when plants are exposed to various abiotic stresses [32,33].

Hussain et al. [34] showed that seed priming with Put was effective in improving seed germination, seedling vigor and enhanced tolerance to drought in maize (Table 1). Seed priming with Spd also improved seed germination under water stress conditions in various species. For example, drought stress experiments performed with white clover seeds revealed that priming with Spd improved germination rate, seeds germinated earlier, and seedlings exhibited enhanced vigor as indicated by longer root length, and higher weights [35]. It was proposed that seed priming with Spd improved starch metabolism, presumably due to elevated α- and β-amylase activities [35]. Seed priming with Spd and Spm in wheat increased the levels of various hormones, accelerated starch degradation, and increased the concentration of soluble sugars during seed germination, which may promote the germination of seeds under drought stress [36]. More recently, it has been reported that seed priming with PAs (especially Put and Spm) modulates drought responses in wheat through the accumulation of osmolytes and the regulation of PA biosynthesis genes expression [37] (Table 1). Sadeghipour [38] showed that seed treatment with PAs (especially combination of Put + Spd + Spm) in mung bean enhances drought tolerance through the accumulation of osmoprotectants, improves water status and reduces oxidative damage. Priming by foliar spraying of PAs in wheat subjected to drought also resulted in better growth, increased levels of photosynthetic pigments, proteins and Rubisco, and improved chloroplast ultra-structure and photosynthetic activity [39]. In addition, wheat plants sprayed with PAs showed clear reductions in electrolyte leakage, Na^+^/K^+^ ratio and ROS markers, as well as elevated catalase (CAT) activity, and reduced ROS levels and malondialdehyde (MDA) [39] (Table 1).

**Table 1:** Exogenous polyamines application induces drought tolerance in different plant species. (*) Experiments based on seed priming.

Plant Species	Polyamine Application	Stress Treatment	Performance	Citations
Citrus	Spm (1 mM) plantets incubated in solution for 5 h	Drought (dehydration for 12 h)	Dehydration tolerance	[27]
Wheat	Put (0.1 mM) foliar spray at the time of anthesis	Drought (witholding water at the time of anthesis)	Drought Tolerance	[22]
Bermudagrass	Spd (5 mM) 21-old-plants in solutions for 7 days	Drought (with holding water for 21 days + 3 days recovery)	Drought Tolerance	[25]
Maize	* Put (0.1 mM) seeds soaked for 10 h	Drought (withholding water for 3 weeks after sowing)	Improved seed germination under water stress	[34]
White clover	* Spd (30 μM) seeds soaked for 3 h	Water stress (PEG 6000) for 7 days	Improved seed germination under water stress	[35]
Tobacco	Put (1 mM) to leaf discs for 2 h	Water stress (PEG 6000) for 1 h	Improved water stress tolerance	[23]
Valerian	Spd, Spm (1 mM) foliar spray at 30 days after transplanting	Drought (witholding water at 30 days after trasnplanting)	Improved drought tolerance	[26]
Wheat	* Spd, Spm, Put (0.1 mM) seeds soaked for 6 h	Water stress (PEG 6000) for 7 days	Improved seed germination under water stress	[36]
Wheat	* Put, Spd, Spm (100 μM) seed soaking for 10 h	Drought (witholding water for 21 days)	Drought tolerance	[37]
Finger Millet	Spd spray (0.2 mM) during 3 weeks at early flowering stage	25, 50 and 75% of water deficits	Alleviate water deficit	[28]
Damask rose	Spd, Spm (0.5 mM) foliar application in plants	50 to 100% water field capacity	Alleviate water deficit	[29]
Lettuce	Put (0.1 mM) foliar application for 8 days in seedlings	Drought PEG 6000 (10%)	Improved drought tolerance	[24]
Mung bean	Seeds soaked in (0 or 100 μM) * Put, Spd, Spm or their mixture for 10 h	In field conditions drought stress (May–August)	Improved seed germination and growth	[38]
Wheat	* Put, Spm and their mixture (100 μM) in seed priming and foliar spray	Drought (withholding water)	Drought tolerance	[39]

### 2.2. Drought Tolerance in Genetically Modified Plants with Altered PA Metabolism 

During the last decades, about 40–50% of gene functions have been experimentally demonstrated in *Arabidopsis* and rice, which has enabled gene annotation and sorting into specific pathways [40,41]. Except for a few regulatory genes, transgenic approaches with individual stress-regulated candidate genes have so far made little impact in plant breeding [42]. By contrast, regulation of the metabolism of osmolytes (for example, Pro) and PAs has emerged as a promising approach to practical applications [40]. In *Arabidopsis*, Put, Spd, Spm and its isomer thermospermine (tSpm) are the most abundant PAs. In this species, arginine decarboxylase (ADC) is the limited step for the synthesis of Put, whereas S-adenosyl-methionine decarboxylase (SAMDC), Spd synthase (SPDS), Spm synthase (SPMS) and Acauliss 5 (Acl5; tSpm synthase) are involved in the formation of Spd, Spm or tSpm [10,12,43].

Characterization of genes encoding PA biosynthetic enzymes from various biological sources and subsequent plant transformation with some of such genes has led to the production of many transgenic plants with improved drought tolerance [44,45,46]. For example, *Arabidopsis* transgenic plants overexpressing the *Arabidopsis ADC2* gene exhibited increased Put and were more drought-tolerant than non-transgenic plants. Interestingly, drought tolerance correlated with the total amount of Put accumulated in the different transgenic lines [47]. ABA-deficient and ABA-insensitive *Arabidopsis* mutants exhibited impaired Put accumulation in response to drought, which indicates that Put accumulation during drought is mainly an ABA-dependent metabolic response [48]. *Arabidopsis* plants with increased Spm levels obtained by overexpression of *SAMDC1* gene also showed enhanced tolerance to drought and salt stress [49]. In contrast, the *Arabidopsis acl5/spms* double mutant, which is unable to produce Spm and tSpm is hypersensitive to drought and saline stress, while exogenous addition of Spm suppressed these phenotypes [50]. A crosstalk between Spm and ABA has also been observed in *SAMDC1* overexpressing *Arabidopsis* plants. These transgenic lines showed elevated levels of ABA due to the induction of *9-cis-epoxycarotenoid dioxygenase* (*NCED*) gene, which encodes a key enzyme involved in ABA biosynthesis [49]. Moreover, *Lotus tenuis* transgenic plants overexpressing the oat *ADC* gene showed a significant increment in Put, and a direct correlation was observed between the *ADC* expression levels and drought tolerance [51]. In addition, the expression of *NCED* was upregulated, suggesting again the existence of an interaction between Put and ABA biosynthesis in response to drought [51]. A remarkable improvement in drought stress tolerance was also observed in tobacco plants overexpressing the *SPDS* gene, along with a reduction in MDA, lower ion leakage and ROS content [52]. Recently, Jiang et al. [53] reported that transgenic *Arabidopsis* plants overexpressing the pear *SPMS* gene exhibited higher Spd and Spm levels, increased Pro content, H_2_O_2_, peroxidase (POD) activity, and soluble sugars in transgenic plants, which displayed improved resistance to both drought and salt stresses [53]. Overall, these results indicate that PAs are key regulators of the homeostasis of antioxidant compounds in plants during drought stress, which gives further support to conclusions obtained from exogenous PA application (see above). 

## 3. Polyamines and Salt Stress

Salinity in soil or water represents one of the most significant abiotic stresses that alter multiple processes in plants. Salt stress strongly depresses germination, growth, development, and productivity of plants worldwide, so that soil salinization and drought pose two of the main problems currently facing agriculture [54]. It is more widespread in arid, semi-arid and coastal regions and progressively increases in irrigated lands. This is due to inappropriate management of irrigation and drainage, low precipitation, high water evaporation and irrigation with saline waters [55]. More than 800 million hectares of land throughout the world are affected by salt. This amount accounts for more than 6% of the world’s total land area. High salinity levels take 1.5 million hectares of land out of production each year [55,56]. Thus, 50% of cultivable lands will be lost by the middle of the 21st century due to salinity [6,57], which affects seed germination through osmotic stress, ion-specific effects, and oxidative stress [58,59]. Water uptake during seed imbibition decreases with increasing external osmotic potential. Salinity can also affect the germination of seeds by the toxic effects from sodium and chloride ions on embryo viability [60]. These toxic effects include disruption of the structure of enzymes and other macromolecules, damage of cell organelles and the plasma membrane, disruption of respiration, photosynthesis, and protein synthesis [60]. Salinity retains water by soil particles, hampering its absorption by plants (osmotic effect) and alters ionic balance, producing nutritional deficiency of K^+^ and other ions, due to elevated concentration of Cl^-^ and Na^+^ (ionic effect) [59].

### 3.1. Protective Effects by Exogenous Application of PAs during Salinity 

Several studies have demonstrated that exogenous applications of PAs improve plant tolerance to salt stress (Table 2). Treatment with Put improved the photosynthetic capacity of cucumber plants by increasing photochemical efficiency of PSII, thus alleviating the deleterious effects of NaCl [61] (Table 2). Quinet et al. [62] reported that exogenous Put reduced Na^+^ accumulation in roots of a salt-sensitive rice cultivar after few days of salt exposure, produced increased Put biosynthesis, and high proportion of conjugated PAs within stressed tissues. Put induced transcriptional activation of genes coding for amine oxidases and increased ethylene production in salt-treated plants [62]. In lemon, Put treatment reduced the salt-induced increase of MDA, suggesting that Put may protect the plasma membrane against stress by maintaining membrane integrity [63] (Table 2). Put was also shown to have a positive effect on photosynthetic machinery of tea plants grown on 50–100 mM NaCl, by controlling ROS scavenging activity [64]. It has also been shown that seed priming with Put is effective at improving seed germination under salinity. For example, Put priming improved seed germination in chamomile and sweet majoram grown under saline conditions [65].

As shown in Table 2, higher PAs appear to be effective in plant protections against salinity-induced damages. Exogenously supplied Spd improved salinity tolerance in cucumber and ginseng seedlings by inducing the activity of antioxidant enzymes and Pro level [66,67]. Similarly, a protective effect of exogenous Spd was observed in two Kentucky bluegrass cultivars through increased activity of several antioxidant enzymes and reduction of MDA levels [68] (Table 2). Treatment with Spd to chrysanthemum seedlings also reduced the uptake of Na^+^, and ameliorated osmotic and ionic balance, enzymatic ROS scavenging capacity, cell membranes stabilization and photosynthetic capacity [69]. Rice seeds soaked with Spd showed improved germination sates and seedling growth by preventing chlorophyll loss, increasing the levels of anthocyanin and phenolics and reducing the contents of H_2_O_2_ and Pro [70] (see Table 2).

Rebecca et al. [71] used A*maranthus* sp. and spinach seeds germinated on Murashige and Skoog (MS) medium supplemented with different concentrations of NaCl (0–50 mM) and Spd (0.01–0.1 mM) to show that Spd improved germination rate as well as protein content. In roots of two cultivars of tomato, the pretreatment with Spd promoted the conversion of free Put into free Spd and Spm, modified the metabolic status of PAs and enhanced tolerance of tomato plants under salinity–alkalinity stress [72]. Shi et al. [25] suggested that PAs could activate multiple pathways that enhance bermudagrass adaption to salinity, which may be applicable for genetically engineering crop plants to improve stress tolerance. In tomato, exogenous Spd application combined with salinity–alkalinity stress decreased the superoxide anion (O_2_-) generation rate and MDA content, as well as increased ascorbate-glutathione cycle components, which resulted in alleviation damage produced by these stresses [73]. In cucumber seedlings, a positive effect of exogenous Spd on photosynthesis associated to improved tolerance to salinity has also been observed [74]. Exogenous Spd treatment also improved salt tolerance in zoysiagrass plants [75]. In sweet sorghum, Spd application alleviated the effect of salt, by enhancing photosynthetic efficiency through regulation of gene expression and activities of key CO_2_ assimilation enzymes [76]. Recently, it has been reported that exogenous Spd application to soybean plants significantly increases sprout growth and biomass by activating the antioxidant capacity in response to salt stress [77]. Furthermore, Jiang et al. [78] showed that application of Spd to rice plants prevented the salt-induced damage in the structure and functions of chloroplasts and content of photosynthetic pigments, by increasing the activities of antioxidant enzymes, thus suggesting that Spd participates in the control of redox homeostasis.

**Table 2:** Exogenous polyamine application induces salinity tolerance in different species. (*) Experiments based on seed priming.

Plant Species	Polyamine Application	Stress Treatment	Performance	Citations
Cucumber	Spd (0.1 mM) 3 days old seedlings for 7 days	Salinity (50 mM NaCl) 3 days old seedlings for 7 days	Salt tolerance enhancement	[66]
Cucumber	Put (65 mM) seedlings with leaf emerging 7 days spray	Salt (65 mM NaCl) seedlings for 8 h	Improved tolerance to salt	[61]
Chamomile	* Put (0.01–1.5 mM) seeds soaked for 10 days	Salinity (25–150 mM NaCl) for 10 days	Improved seed germination under salinity	[65]
Sweet majoran	* Spd (0.01–1.5 mM) seeds soaked for 10 days	Salinity (25–150mM NaCl) for 10 days	Improved seed germination under salinity stress	[65]
Spinach	* Spd (0.01–0.1 mM) seeds soaked	Salinity (50 mM NaCl)	Improved seed germination under salinity	[71]
Amarathus	* Spd (0.01–0.1 mM) soaked	Salinity (50 mM)	Improved seed germination under salinity	[71]
Rice	Put (1 mM) 10 days old seedlings for 12 h	Salinity (100 mM NaCl) 10 days old seedlings for 12 h	Reduced Na^+^ accumulation in salt sensitive cultivars	[62]
Tomato	* Spd (0.25 mM) seeds soaked for 10 h	Salinity-alkalinity solution	Improved tolerance	[72]
Bermudagrass	Spd (5 mM) 21 old plants solution for 7 days	Salt (50–300 mM NaCl) to 28 days old plants for 24 h	Salt tolerance	[25]
Ginseng	Spd (1 mM) 2-week-old seedlings for 7 days	Salt (150 mM) for 7 days	Salt tolerance enhancement	[67]
Lemon	Put (1 mM) foliar spray for 2 weeks to 2-month-old plants	Salt (25–100 mM NaCl) for 2 weeks	Enhanced salt tolerance	[63]
Rice	* Spd (1 mM) soaked seeds for 14 days	Salinity (150 mM NaCl) for 10 days	Salt tolerance enhanced	[70]
Tomato	Spd (0.25 mM) foliar spray to seedlings for 8 days	Salinity-alkalinity solution	Enhanced tolerance to stress	[73]
Bluegrass	Spd (1 mM) 2 week-old-seedlings for 7–28 days	Salt (50–200 mM NaCl) gradual increment during 7–28 days	Salt tolerance enhancement	[68]
Chrysantemum	Spd (0.5–2 mM) 4 foliar application 20 days old	Salt (NaCl 75 mM) 6 days old seedlings with 3 leaves	Salt tolerance enhancement	[69]
Cucumber	Spd (0.1 mM) for 6 days to seedlings with 3 leaves	Salt (75 mM) to seedlings with 3 leaves	Salt tolerance enhancement	[74]
Zoysiagrass	Spd (0.15 mM) in two cultivars with high and lower salinity tolerance	NaCl (150 mM) and mix (Spd and NaCl) from 0–8 days	Salt tolerance enhancement	[75]
Tea	Put (5 mM) in plants of 2-years-old and 7–8 leaves on bud foliar application	Put (5 mM) + NaCl (50–100 mM) during 1–8 days	Alleviating salt-stress	[64]
Sweet sorghum	Spd (0.25 mM) in Hoagland solution in 10 days-old-seedlings	NaCl (100–150 mM) and mix (NaCl and Spd)	Enhanced photosynthetic efficiency	[76]
Soybean	* Spd (0.10 mM) in soaked seeds from 4–6 days	Salt (50 mM NaCl) and Mix (Spd + NaCl)	Alleviated salt stress	[77]
Rice	Spd (from 0–1.5 mM), 7 days treatment, and 4th fully expanded leaves	NaCl (100 mM) and mix (NaCl + Spd)	Stability of chloroplasts against salt stress	[78]

### 3.2. Protective Effects against Salinity in Genetically Modified Plants with Altered PA Metabolism 

Salt tolerance can be achieved by overexpression of genes encoding several PA biosynthetic enzymes in various species [17,18]. For example, Roy and Wu [79] found that the stress-inducible expression of the *ADC* gene resulted in increased ADC activity and overproduction of Put and total PA levels in transgenic rice plants. It was suggested that increased PA levels contributed, at least in part, to the enhanced biomass production in transgenic rice plants under saline stress conditions [79]. This is consistent with the results obtained in an *adc2 Arabidopsis* mutant, in which free Put content was reduced as compared to the control plants and Put levels did not increase under salt stress. The *adc2* mutant was more sensitive to salt stress than wild type, and stress sensitivity of the mutant was recovered by the addition of exogenous Put, thus suggesting that *ADC2* expression is required for Put accumulation in salt tolerance [80]. Under salt stress, accumulation of Spd and Spm in transgenic rice overexpressing *SAMDC* may also contribute to increased stress tolerance as shown by the increased biomass production as well as increased shoot length of the transgenic lines compared to the wild type [81]. These results are in agreement with the enhanced salt tolerance observed in *Arabidopsis* and tobacco transgenic plants overexpressing *SAMDC* [13,49,50]. In the same line, Alet et al. [82] showed that deletion of *SPMS* and *tSPMS* genes led to higher levels of the toxic Na^+^ ion and reduced growth under salinity conditions. Furthermore, overexpression of the apple *SPDS1* gene in pear led to Spd accumulation and improved stress tolerance against many stresses including salinity, which was associated with the activation of antioxidant enzyme activities and reduction in the concentration of MDA [83]. Yamaguchi et al. [84] studied the role of Spm during high salt stress using an *Arabidopsis* double knockout-mutant plant (*acl5/spms*) which cannot produce Spm. This Spm-deficient mutant was hypersensitive to high salt stress and this phenotype was abrogated by exogenously applied Spm. Furthermore, this mutant plant exhibited a symptom of Ca^2+^ deficiency [84]. Based on the results obtained, these authors proposed that salt-treated plants enhanced PA biosynthesis and the resulting higher level of Spm modulated the activity of certain Ca^2+^-permeable channels. This in turn would result in (i) prevention of Na^+^/K^+^ entry to the cytoplasm, (ii) enhancement of Na^+^/K^+^ influx to the vacuole, or (iii) suppression of Na^+^/K^+^ release from the vacuole [84].

Accumulation of PAs in plants subjected to salt stress not only depends on PA biosynthesis, but also PA catabolism. The *Arabidopsis* polyamine oxidase five (*AtPAO5)* is the *PAO* gene member most transcriptionally induced by salt stress [85]. Two independent loss-of-function mutants (*atpao5-2* and *atpao5-3*) were found to exhibit constitutively higher tSpm levels, which were associated with increased salt tolerance. Stimulation of ABA and jasmonate (JA) biosynthesis and accumulation of important compatible solutes, such as sugars, polyols and Pro, as well as TCA cycle intermediates were observed in *atpao5* mutants under salt stress. Expression analyses indicated that tSpm modulated the transcript levels of several target genes (i.e., those involved in JA biosynthesis and signaling), some of which are already known to promote salt stress tolerance. Overall, it was concluded that tSpm triggers metabolic and transcriptional reprogramming that promotes salinity tolerance in *Arabidopsis* [85].

## 4. Polyamines and Low Temperature Stress

All plant species have an optimum temperature range for efficient physiological functions. Temperatures above or below that range have a negative impact on plant performance, which leads to a loss in economic yield. Chilling and freezing are collectively known as cold- or low-temperature stress. Chilling stress is induced when temperatures are below the optimum and low enough to cause injury without producing ice crystals within the soft tissues of the plants. Freezing stress occurs when ice crystals are produced in soft tissues [86]. Tolerance capabilities for chilling (0–15 °C) and freezing (<0 °C) temperatures vary among different species. Chilling occurs regularly in temperate and tropical plants, which are rarely exposed to freezing conditions. Survival of rice, maize and soybean plants is compromised by temperatures below 0 °C, whereas wheat and barley show several degrees of freezing tolerance [18]. Cold acclimation is an adaptive process that some species from temperate regions have developed in response to low, non-freezing conditions. Numerous molecular, biochemical, and physiological changes occur during cold acclimation [86], most of them being associated with significant changes in gene expression and metabolite profiles including PAs [7].

### 4.1. Protective Effects by Exogenous Application of PA during Low Temperature Stress

Several studies have reported that exogenous PA treatments improve plant tolerance to low temperature [14] (see also Table 1). For example, Put-priming of fennel seeds improved germination performance and seedling growth and enhanced tolerance to low temperature stress, as compared with non-primed seeds [87] (Table 3). In tomato, exogenous Put improved tolerance to chilling by reduction of H_2_O_2_ and MDA levels and modulation of the antioxidant machinery [88]. More recently, the involvement of ABA has been reported in the Put-induced tolerance to chilling stress [89], which is consistent with previous results obtained in *Arabidopsis* [90]. Abbasi et al. [91] investigated the effects of Put application (spray inoculation) on the quality characteristics of peach fruit during low-temperature storage. The results showed that Put significantly reduces the rate of fruit softening, loss in fruit weight, total soluble solids (SSC), titratable acidity (TA), ascorbic acid content and fading of skin colour during storage, regardless of the doses of Put applied, or the time of application.

Regarding Spd, cucumber plants pre-treated with this polyamine before they were exposed to chilling showed higher growth rates and leaf chlorophyll content than control plants during chilling [92]. Moreover, pre-treatment with Spd alleviated the decline of chlorophyll fluorescence yield, the photosynthetic electron transfer activity of thylakoids, and the activity of various enzymes involved in C metabolism, and reduced lipid peroxidation in the thylakoid membranes. Overall, the results indicated that Spd pre-treatment improved chilling tolerance of the photosynthetic apparatus in cucumber [92]. Exogenous pre-treatment with Spd also alleviated low temperature injury in mung bean seedlings by modulating of the ascorbate–glutathione pathway and reduction of components in the glyoxalate cycle, indicating that oxidative stress was reduced in Spd pre-treated seedlings [93]. In rice, it has recently been reported that seed priming with Spd improves tolerance to chilling stress by increasing α-amylase activity, soluble sugars and protein contents, as well as the activity of antioxidant systems [94] (see Table 3). Physiological responses to low temperature were also analyzed by Chen et al. [95] in a chilling-tolerant centipedegrass (*Eremochloa ophiuroides*). Exogenous Put or Spd applications increased antioxidant enzyme activities and chilling tolerance, suggesting that PA regulation of antioxidant systems is important for chilling tolerance. Jankovska-Bortkevič et al. [96] found that exogenous PA application (Put, Spd and Spm) in winter oilseed rape maintained the activity of plasma membrane H^+^-ATPase, increased the content of Pro, and delayed the stimulation of ethylene emission under increasing cold conditions. Therefore, PAs may act as elicitors that activate a stress protection response, which may compensate for the negative impact of low temperature stress. 

### 4.2. Protective Effects to Low Temperature Stress in Genetically Modified Plants with Altered PA Metabolism

The first study which described the involvement of PAs in plant freezing tolerance using transgenic approaches was reported in *Arabidopsis* plants transformed with the *SPDS* cDNA from *Cucurbita ficifolia* under the control of the CaMV 35S promoter. *SPDS* over-expressor lines exhibited higher SPDS activity, together with an increase in PA levels (mainly Spd and Spm). *SPDS* transgenic lines were more tolerant to several abiotic stresses, including chilling and freezing, than the wild-type plants [97].

Knock-out mutants for *Arabidopsis ADC1* are more sensitive to freezing, but this phenotype can be reversed by exogenous application of Put [98]. We have also shown that constitutive overexpression of *Arabidopsis ADC1* increases endogenous Put and promotes freezing tolerance in both *Arabidopsis* and tobacco plants [99]. In *Arabidopsis*, increased freezing tolerance was also observed in acclimated and non-acclimated *ADC1* over-expressor lines, although differences were more striking under cold acclimation [99]. Increased tolerance to freezing was also observed in *Arabidopsis* transgenic plants overexpressing the oat *ADC* gene under the control of the *RD29A* stress-inducible promoter [100]. Wi et al. [101] studied transformed tobacco plants with the carnation *SAMDC* cDNA under the control of the CaMV 35S promoter. These lines accumulated higher levels of Put, Spd and Spm. Under control conditions, transgenic *SAMDC* tobacco lines were healthy and only small differences in size and flowering time were apparent compared to wild-type plants. These lines showed enhanced tolerance not only to low temperature stress, but also to salt, acidic and oxidative stress, as well as to ABA treatment [101]. Recently, an association has been found between cold tolerance and PAO mediated H_2_O_2_ production, which in turn leads to nitrate reductase (NR)-derived nitric oxide (NO) production and induced antioxidant enzyme activities in transgenic centipedegrass plants [102]. In conclusion, the different transgenic approaches described by several works demonstrate the feasibility to improve tolerance against low temperature stress by PA overproduction [7,10,18].

## 5. Polyamines and Heat Stress

The constantly rising ambient temperature is considered one of the most detrimental stresses. The global air temperature is predicted to rise by 0.2 °C per decade, which will lead to temperature increases of 1.8–4.0 °C by 2100 [103]. Temperatures above the optimum range for plant growth and development can injure or permanently damage both vegetative and reproductive organs, thus limiting crop productivity worldwide. Plant responses to heat (HT) vary with the degree and duration of HT and the plant type [104]. A plant is able, to some extent, to tolerate heat stress by physiological changes and metabolism adaptation [41]. Plants alter their metabolism in various ways in response to HT, particularly by producing compatible solutes that are able to organize proteins and cellular structures, maintain cell turgor by osmotic adjustment, and modify the antioxidant system to re-establish the cellular redox balance and homeostasis [105]. At the molecular level, heat stress causes alterations in the expression of genes, including those responsible for the expression of osmoprotectants, detoxifying enzymes, transporters, and regulatory proteins [41]. Modification of physiological and biochemical processes by changes in gene expression gradually leads to the development of heat tolerance in the form of acclimation, or in the ideal case, adaptation to HT [103]. In recent years, exogenous application of osmoprotectants (Pro, glycine betaine, trehalose, etc.), phytohormones (abscisic acid, gibberellic acids, jasmonic acid, etc.), signaling molecules (e.g., nitric oxide, NO) and PAs have been found to be effective in mitigating HT stress-induced damage in plants [106,107].

### 5.1. Protective Effects Produced by Exogenous PA Application Against Heat Stress

The effect of exogenous PA treatment on plant tolerance to HT has been the subject of many studies [15] (see also Table 3). For instance, the effect of Put, Spd and Spm in heat-shock protection was investigated in soybean seedlings [108]. Pre-treatment with PAs 2 h before heat-shock at 45 °C for additional 2 h, enhanced the recovery of both roots and hypocotyl growth. It was shown that PAs decreased electrolyte leakage and MDA levels from different tissues, thus suggesting protection of membrane integrity. The results also suggested that under stress conditions, PAs may replace Ca^2+^ in maintaining membrane integrity by binding to membrane phospholipids [108]. The exposure of wheat plants to HT stress (35 °C) during 4 h or 8 h significantly decreased growth, total PA and essential amino acid contents [109]. Pre-treatment of wheat plants with Put before exposure to HT led to higher tolerance to heat stress possibly by increasing total PA and amino acids contents, and decreasing ethylene and NH_4_^+^ (considered as a very toxic product) production [109]. Genome-wide expression profiles of tomato fruits following their exposure to HT and exogenous Spd application (which alleviates HT injury) was studied by Cheng et al. [110]. Under normal temperature, the Spd-regulated genes were quite different as compared to stress-related genes in response to HT. However, under HT conditions, when fruits were pretreated with exogenous Spd, the number of genes involved in signal transduction was significantly increased. Many regulatory factors, ethylene-related genes, PA biosynthetic genes, hormone pathways genes, and oxidation reduction genes exhibited regulation in response to Spd treatment. The results indicated that Spd might play an important role in the regulation of tomato fruit response to HT during ripening stage [110]. More recently, proteomic approaches have also been used to investigate the effects of exogenous Spd to tomato seedlings exposed to HT [111]. A total of 67 differentially expressed proteins were identified in response to HT and/or exogenous Spd. Exogenous Spd up-regulated most identified proteins involved in photosynthesis, implying an enhancement in photosynthetic capacity. Physiological analyses showed that Spd could improve net photosynthetic rate and biomass accumulation. In addition, the results suggested that increased heat-stress tolerance by exogenous Spd could contribute to the higher expression of proteins involved in cell rescue and defense, and that Spd may regulate the antioxidant enzyme activities and related genes expression in tomato seedlings exposed to HT [111]. Recently, Jing et al. [112] showed that exogenous Spd and Spm in spring wheat increased the relative water content, chlorophyll levels, stomatal conductance, transpiration rate, the maximal quantum yield of PSII photochemistry, antenna conversion efficiency, and photochemical quenching coefficient of flag leaves under HT.

Jing et al. [113] measured the endogenous PA levels during grain filling in two wheat varieties differing in heat resistance. It was found that grain weight is negatively correlated with the content of Put, MDA, Pro, and SOD and CAT activities, but positively correlated with the content of Spd/Spm and activity of POD in grains, indicating that the exogenous Spd and Spm could alleviate heat injury during grain filling.

High temperature and drought stresses often occur simultaneously, and in the context of global climate change, this stress combination exerts devastating effects on plants. In this regard, it has been shown that Spm pretreatment can enhance the tolerance of trifoliate orange plants to combined HT and drought [114] (Table 3). Exogenous application of Spm allows the plants to maintain higher levels of antioxidant enzyme activities, stronger scavenging of ROS and higher expression of stress-related genes under combined stress conditions. Spermine also reduces lipid peroxidation and damage to cell membranes and prevents stress-induced protein denaturation and aggregation to protect the trifoliate orange seedlings from stress damage. Thus, this study provides evidence supporting that PAs confer tolerance to multiple and simultaneous stresses [114]. More recently, Nahar et al. [115] reported that Spm pretreatment enhanced the tolerance of mung bean plants to HT, drought, and combined HT and drought stresses. Spermine significantly reduced the generation of ROS, lipoxygenase activity, and lipid peroxidation as well as enhancing the activity of antioxidant enzymes. Maintenance of plant water status is vital for HT and/or drought tolerance. In this sense, it was shown that exogenous Spm regulates the level of Pro and maintained a good water status of mung bean seedlings. The overall tolerance of plants was shown from protection of photosynthetic pigments and prevention of reduction of leaf area which are vital for photosynthesis. All of this resulted in the improved growth, biomass production and phenotypic appearance of mung bean seedlings. Thus, this study provided additional evidence supporting that PAs confer tolerance to multiple and simultaneous stresses [115].

**Table 3:** Exogenous polyamines application induces low- and high-temperature tolerance in different species. (*) Experiments based on seed priming.

Plant Species	Polyamine Application	Stress Treatment	Performance	Citations
Cucumber	Spd (0.5 mM) second leaf fully expanded for 2 days	Chilling (10/7 °C) for 8 days returned at (28/22 °C) for 3 days	Enhaced tolerance to low temperature	[92]
Fennel	* Put (10–20 ppm) for 24 h soaked seeds	Chilling (10 °C)	Enhanced tolerance to low temperature	[87]
Mung bean	Spd (0.25 mM) pretreated 4 days old seedlings for 24 h	Low temperature (6 °C) for 2–3 days	Enhanced tolerance to low temperature	[93]
Tomato	Put (1 mM) foliar spray, one for a week to 5-leaf seedlings	Chilling (4 °C) for 24 h and recovery (25/15 °C) for 10 days	Enhanced tolerance to low temperatures	[89]
Rice	* Spd (5 mM) for 24 h soaked seeds	Chilling (10 °C)	Enhanced chilling tolerance	[94]
Tomato	Put (1 mM) in 5 th leaf stage	Chilling (10 °C)	Enhanced tolerance to chilling	[88]
Centipedegrass	Put or Spd (0.1 mM) solution/pot in plants of 60 days	Chilling (8 °C) for 30 days, analysis of the 3rd leaf from the top	Enhanced tolerance to chilling	[95]
Peach	Put (0, 1, 2, 3 mM) in 3 stages of fruit development	Storage of fruits at (1 °C) for 6 weeks	Chilling injury alleviated	[91]
Oilseed rape	Put, Spd, Spm (1 mM) foliar spray in plants cultivated 22 days	Cold acclimation, 4 days (4 °C) and two d increasing cold (from −1 to −3 °C)	Improved cold resistance	[96]
Soybean	Put, Spd (1 mM) pretreatment of germinating seedlings 2 h	(45 °C) for 2 h	Enhanced tolerance to heat	[108]
Wheat	Put (2.5 mM) foliar spray 30 days old seedlings	Heat (35 °C) for 4–8 h	Enhanced tolerance to heat	[109]
Tomato	Spd (1 mM) immersion of green fruits 30 min	Heat (37/27 °C) for 1–12 h	Enhanced tolerance to heat	[110]
Tomato	Spd (1 mM) 3rd true leaf foliar spray	Heat (28/38 °C), 7 days	Enhanced tolerance to heat	[111]
Trifoliate orange	Spm (1 mM) 3-months-old seedling 30 h pretreatment	Heat (45 °C) for 180 min produced HT and dehydration	Enhanced tolerance to heat and drought	[114]
Mung bean	Spm (0.2 mM) 5 days old seedlings pretreatment for 24 h	Heat (40 °C) + drought 0.5% PEG 6000 for 48 h	Enhanced tolerance to heat and drought	[115]
Wheat	Spd, Spm (1 mM) sprayed before HT and lasted for 5 d on the flag leaves and panicles	In the field: Average temperature inside (34.9 °C) and outside (30.5 °C)	Alleviate the photosynthesis in flag leaves	[112]
Wheat	Spd, Spm (1 mM) before HT, sprayed on the flag leaves and panicles	In the field: otuside the shed (26.7–32.3 °C) and inside the shed (37.7–32.1 °C)	Alleviate grain filling	[113]

### 5.2. Protective Effects to High Temperature Stress in Genetically Modified Plants with Altered PA Metabolism

Improved thermotolerance resulting from over- or under-expression of PA metabolism genes has been reported in various species [17,44]. For example, the expression profile of PA biosynthetic genes in response to HT in *Arabidopsis* revealed that *SPMS*, *SAMDC2* and *ADC2* expression are up-regulated during the early stages of heat stress, which is accompanied by elevated Put, Spd and Spm levels [116]. By using transgenic *Arabidopsis* plants overexpressing *SPMS* and mutants deficient in Spm, a direct correlation between high endogenous levels of Spm and improved plant thermotolerance was observed [116]. In tomato, thermotolerance could be achieved in transgenic plants overexpressing the yeast *SAMDC* gene, which caused significant increases in Spd and Spm levels [117]. It was suggested that the enhanced tolerance to HT in the tomato transgenic plants was due to increased levels of antioxidant activities and protection of membrane lipid peroxidation [117]. More recently, genetically modified tobacco plants over- or under-expressing the *ZmPAO* gene were studied under heat stress. Lower expression of *ZmPAO* correlated with increased thermotolerance of the photosynthetic machinery and improved biomass accumulation, which was accompanied by enhanced levels of enzymatic and non-enzymatic antioxidants. Conversely, *ZmPAO* overexpression lines were compromised in thermotolerance. These results provide important clues about the involvement of PA catabolism and H_2_O_2_ generation in thermotolerance [118]. Overall, these studies together with the results discussed in the previous section indicate that PAs are key players in the regulation of plant thermotolerance.

## 6. Final Remarks and Future Directions

A significant number of studies report improved stress tolerance by using either exogenous PA applications or genetic manipulation of endogenous PA levels in transgenic plants. There are already several patents on the use of polyamines as stress-protective compounds (see for example [99]). However, we still do not know the precise molecular mechanism underlying PA protective effects against stress. The classical idea of PAs as molecules with a protective role comes from their chemical structure. Polyamines have a polycationic nature that makes them able to participate in the modulation of the cell ion balance and binding to polyanionic molecules such as DNA, RNA, proteins or membrane lipids by preventing macromolecule degradation and protecting cell membranes from damages produced by stress. The binding properties of PAs might confer radical-scavenging capacity, which suggests the possibility of an antioxidative role for PAs by inhibition of lipid peroxidation and ROs production. Additionally, one of the products of PA oxidation is H_2_O_2_, which can act in stress signaling [10,12].

The relatively recent use of modern molecular and genetic techniques has provided some evidence that PAs could act as signaling molecules [10,12]. In this sense, it has been shown that modification of endogenous PA levels triggers transcriptional changes resembling stress activation [49]. Such response might involve ROS production but also cross-modulation with other hormones. Indeed, several reports point to a crosstalk between PAs and ABA through stimulation of ABA biosynthesis by PAs [98] and PA biosynthesis by ABA [48]. Another possible explanation for some of the transcriptional changes observed in PA overexpressors plants could be the existence of a plant PA modulon expression system, such as was first discovered in *Escherichia* *coli*, which is composed by several transcriptions factors stimulated by PAs at the translational level [119]. The study of plant PA signaling is and active area of research which might also lead to practical applications.

On the other side, it has been shown that PAs affect physiological responses such as stomata aperture [120]. A model has been proposed to explain the involvement of PAs on the regulation of stomatal closure where PAs participate through H_2_O_2_ produced by its oxidation, as well as by their interaction with nitric oxide (NO) signaling [121]. In this sense, PAs could act synergistically with ROS and NO in promoting ABA responses in guard cells [12]. Furthermore, it has also been suggested that there is a possible link between PAs, Ca^2+^ homeostasis and stress responses [12]. It has been proposed that Spm could exert a protective role against salinity and drought conditions through the control of Ca^2+^ allocation by regulation of Ca^2+^ permeable channels. Thus, a well-organized protection mechanism comprising of PAs, Ca^2+^, ABA, H_2_O_2_ and NO seems to coordinate an adapt the response of plants to abiotic stress [10,12]. Remarkably, the salt-tolerant *pao5* mutant accumulates T-Spm and triggers ABA-and JA-dependent signaling contributing to salt tolerance [85], which adds further complexity to PA cross-talks with other components of stress signaling.

In the future, we need to deeply study PA signaling and early events triggered by these compounds. We still need to address fundamental questions such as PA transport between organelles and cells, PA perception and signaling pathways. We envisage that a detailed mechanistic and signaling analysis addressing these and other fundamental questions will provide new leads for crop protection against biotic and abiotic stresses.

In recent years, molecular priming has become an interesting and cost-effective approach to inducing plant tolerance. Thus, structurally unrelated organic molecules have been shown to prime plants against stress [122]. As indicated in previous sections, seed priming with PAs (Put, Spd and Spm) results in improved tolerance to abiotic stress (drought, salt, low and high temperatures), as well as improved growth performance of germinating seedlings. Polyamines have the advantage of being naturally occurring compounds, which are non-toxic to plants and mammals at the effective concentrations applied. Therefore, PAs can be used for stress mitigation and to increase crop yield and quality without any negative effect for crops or the environment. The use of PA seed priming against multiple stresses in many different crops (see previous sections) might find future practical applications for crop protection against stress.
